# 2255: Prevalence and management of chronic pain syndromes during pregnancy

**DOI:** 10.1017/cts.2017.101

**Published:** 2018-05-10

**Authors:** Shona Ray-Griffith, Bethany Morrison, Pedro Delgado, Everett Magann, Michael Mancino, Zachary Stowe

## Abstract

OBJECTIVES/SPECIFIC AIMS: (1) Characterize the prevalence and initial pharmacological management of chronic pain syndromes during pregnancy in a women’s mental health program. (2) Describe the severity and qualitative characteristics of chronic pain during pregnancy and the acute postpartum period. (3) Compare obstetrical and neonatal outcomes between pregnant women with and without chronic pain syndromes. METHODS/STUDY POPULATION: A chart review was conducted to identify all pregnant women who presented for an initial evaluation to the Women’s Mental Health Program (WMHP) at the University of Arkansas for Medical Sciences from July 2013 to June 2016. We excluded respondents <18 years of age or who did not consent to having their information used for research purposes. Demographic information, past and current medical histories, and medication history were obtained from written and electronic medical records. Chronic pain complaints and medication history are presented as counts and percentages. In an ongoing prospective, longitudinal study of pregnant women with chronic pain, women are enrolled before 20 weeks gestation and followed throughout pregnancy and the first 3 months postpartum. Study visits occur at 4-week intervals; and pain characteristics, analgesic exposures, other medications, and depressive measures are collected. Obstetrical and neonatal outcomes are obtained following delivery. Subjects will be compared based on pain types (ie, neuropathic pain, non-neuropathic pain, and controls) and treatment exposures (eg, +/− opioids). Primary outcome measures include visual analog scale (VAS). Secondary outcome measures include other pain and depression assessments. Data will be analyzed using SAS 9.4. A *p*-value of<0.05 was considered statistically significant. RESULTS/ANTICIPATED RESULTS: (1) Chronic pain conditions were reported by 28.2% (44/156) of the initial referrals to the WMHP. (2) 95.5% of respondents with chronic pain were taking at least 1 medication, and 59.5% were taking 2 or more medications. Mean number of medications used were 2.6±2.1.3. The most common medications reported were acetaminophen (43.2%), opioids (43.2%), and sedative/hypnotics (36.4%). Non-pharmacological therapy (eg, physical therapy and transcutaneous electrical nerve stimulation) was reported by 20.5% of respondents. (4) We anticipate that measures of pain severity will increase in pregnancy, peak in the third trimester, and decline in the postpartum period. (5) We foresee that the prospective results will confirm the chart review as indicated by a higher rate of medication exposures during pregnancy, including non-analgesic medications in the women with chronic pain syndromes. (6) We expect women with chronic pain syndromes to have a higher rate of obstetrical complications, specifically pre-term delivery and operative delivery. (7) Finally, we anticipate that chronic pain syndromes and management will result in a higher rate of neonatal complications, specifically neonatal intensive care unit admission, neonatal respiratory problems, and small for gestational age infants. DISCUSSION/SIGNIFICANCE OF IMPACT: Chronic pain syndromes are prevalent in more than one-quarter of pregnant women in our study with the majority of women using pharmacological agents to manage their condition. This prevalence is greater or equal to than other common obstetrical conditions, such as gestational diabetes or preterm delivery. The novel prospective data will be germane to the clinical care of pregnant women with chronic pain disorders. Clinical practice will be better informed by our data regarding the potential impact of chronic pain and its management on pregnancy course and perinatal outcomes. These data will provide the initial foundation for the development of treatment guidelines for the management of chronic pain syndromes during the perinatal period.

